# SARS‐CoV‐2 Infection Is Associated With an Increased Risk of Hospital‐Treated Infectious Mononucleosis due to EBV: National Register‐Based Cohort Study

**DOI:** 10.1002/jmv.70787

**Published:** 2025-12-29

**Authors:** Snieguole Vingeliene, Huiqi Li, Helena Backman, Ruzan Udumyan, Johan Jendeberg, Gunlög Rasmussen, Martin Sundqvist, Marleen A. H. Lentjes, Katja Fall, Ayako Hiyoshi, Fredrik Nyberg, Scott Montgomery

**Affiliations:** ^1^ Clinical Epidemiology and Biostatistics School of Medical Sciences, Faculty of Medicine and Health Örebro University Örebro Sweden; ^2^ School of Public Health and Community Medicine, Institute of Medicine, Sahlgrenska Academy University of Gothenburg Gothenburg Sweden; ^3^ Department of Obstetrics and Gynaecology Faculty of Medicine and Health Örebro University Örebro Sweden; ^4^ Department of Radiology Faculty of Medicine and Health Örebro University Örebro Sweden; ^5^ Department of Infectious Diseases School of Medical Sciences, Faculty of Medicine and Health Örebro University Örebro Sweden; ^6^ Department of Laboratory Medicine, Clinical Microbiology Faculty of Medicine and Health Örebro University Örebro Sweden; ^7^ School of Medical Sciences, Faculty of Medicine and Health Örebro University Örebro Sweden; ^8^ The Institute of Environmental Medicine Karolinska Institutet Stockholm Sweden; ^9^ Department of Medicine Clinical Epidemiology Division Solna, Karolinska Institutet Stockholm Sweden; ^10^ Department of Epidemiology and Public Health University College London London UK

**Keywords:** Epstein‐Barr virus, infectious mononucleosis, register study, SARS‐CoV‐2

## Abstract

There is evidence that persistent dysregulation of the immune system caused by SARS‐CoV‐2 infection may increase susceptibility to other infections. Here, we assessed whether it is associated with subsequent diagnoses of infectious mononucleosis due to Epstein‐Barr virus (EBV‐IM). Residents of Sweden aged 3–100 years without a prior diagnosis of EBV‐IM were followed between January 1, 2020, and November 30, 2022, comprising a total of 9 978 860 participants. Individuals were categorized into those without a COVID‐19 diagnosis, those with a positive SARS‐CoV‐2 polymerase chain reaction (PCR) test only – less severe exposure, and those admitted to hospital with COVID‐19 – more severe exposure. Cox regression was used to estimate hazard ratios (HR) with 95% confidence intervals (95% CI) for the association between the exposure, modeled as a time‐varying covariate, and EBV‐IM occurrence. EBV‐IM rates per 100 000 person‐years and 95% CIs were 4.6 (4.4–4.9) for individuals not diagnosed with COVID‐19, 7.8 (6.9–8.9) for those with a positive SARS‐CoV‐2 test only, and 10.5 (6.2–17.6) for patients admitted to hospital with COVID‐19. HR and 95% CI were 1.61 (1.39–1.88) for people with a positive PCR test only and 5.71 (3.33–9.79) for those admitted to hospital with COVID‐19 compared with people without a COVID‐19 diagnosis, after adjustment for birth year, sex, Swedish healthcare region, region of birth, and Charlson comorbidity index. SARS‐CoV‐2 infection was associated with a subsequent raised risk of EBV‐IM, including among those with less severe acute infection, signaling immune perturbation and the possibility of further delayed sequelae linked with EBV‐IM.

## 1 Introduction

The international coronavirus disease (COVID‐19) pandemic that began in 2020 was caused by the severe acute respiratory syndrome coronavirus 2 (SARS‐CoV‐2). We hypothesized that COVID‐19 may be associated with a raised risk of a subsequent diagnosis of the symptomatic variant of Epstein‐Barr virus (EBV) infection: infectious mononucleosis (IM), which is an important risk factor for other diseases [1, 2, 3, 4, 5]. Evidence of persistent consequences of COVID‐19 includes post‐acute infection symptoms with neurological and psychiatric symptoms, such as fatigue, headache, and attention disorder, that persist in a significant proportion of individuals, lasting weeks or months after the initial diagnosis of the infection [6]. Post‐infectious fatigue syndrome is not unique to SARS‐CoV‐2 infection and has previously been reported after other infections such as influenza and EBV [7], indicating that the lack of recovery may be due to long‐lasting dysregulation of the immune response. A recent scoping review of 23 studies of post‐COVID‐19 patients suffering from persistent symptoms, including fatigue, dyspnea, myalgia, and sleep disorders, identified a set of biomarkers related to the immune response, including T‐lymphocytes CD4+, CD8+, interleukin (IL)‐6, and tumor necrosis factor alpha (TNF‐α) [8]. Post‐infection dysregulation of the immune response has been demonstrated at transcriptional, serological, and cellular levels until at least 6 months after a positive SARS‐CoV‐2 test [9]. An increased susceptibility to other infections during these initial months after COVID‐19 is possible, as there have been reports of different types of secondary infections, including viral, bacterial, and fungal [10, 11]. However, it is unknown if infection with SARS‐CoV‐2 may specifically lead to an increased risk of IM caused by EBV, commonly known as glandular fever, due to persistent immune perturbation.

EBV infection is common, and over 90% of the world's population is thought to have an established lifelong infection with EBV by the age of 30 years [12]. EBV infection in younger children is usually asymptomatic, whereas infection in adolescence or early adulthood can present as infectious mononucleosis (EBV‐IM), which may develop into a severe acute illness requiring hospital admission [13]. EBV‐IM is an important risk factor for MS [1, 2], and there is convincing evidence for a long‐term risk of developing MS in a minority of those with some patterns of seropositivity for EBV infection [14, 15, 16]. Some patterns of EBV seropositivity are also associated with other severe but rare sequelae, including systemic lupus erythematosus, Sjögren syndrome, and rheumatoid arthritis, as well as various cancers, including Hodgkin's lymphoma, Burkitt's lymphoma, and nasopharyngeal cell carcinoma [4, 13]. The symptomatic manifestation of EBV as infectious mononucleosis potentially confers an even greater risk for disease development, compared with EBV seropositivity alone [17, 18], and has been associated with delayed sequelae, including diseases such as Hodgkin's lymphoma [5], and possibly inflammatory bowel disease [19]. Reactivation of EBV has been reported in patients admitted to intensive care [20] as well as during long COVID‐19 [21, 22]. However, only a few case studies have reported IM in COVID‐19 patients [23, 24]. EBV‐IM following SARS‐CoV‐2 could indicate an atypical immune response that confers a raised risk of subsequent delayed sequelae.

We conducted a national register study in Sweden to examine the association between recorded SARS‐CoV‐2 infections and subsequent risk of an EBV‐IM hospital diagnosis. A greater severity of COVID‐19 was indicated by hospital admission.

## 2 Methods

### 2.1 Study Population and Data Sources

Data from the SCIFI‐PEARL (Swedish COVID‐19 Investigation for Future Insights – a Population Epidemiology Approach using Register Linkage) project [25] were used for this study. SCIFI‐PEARL was established to facilitate more efficient research on COVID‐19 [25] by linking multiple national registers that recorded information prospectively. The registers were linked by the pseudonymised Swedish personal identification number, which is a mandatory identifier assigned to each resident in Sweden. The type of register linkage is deterministic, as there are no missing personal identification numbers, and there are no temporal gaps, as the information is reported prospectively to the registers continuously. The most recent data update was completed on November 30, 2022, aligning with the end of the follow‐up period of the study. In this cohort study, data from the following registers were used: The Total Population Register (TPR), the National Patient Register (NPR), SmiNet, the Intensive Care Register, and the National Vaccination Register (NVR) (Figure SI).

The TPR provided information on sex, dates of birth, region of residence, migration dates, and death. The NPR was used to obtain EBV‐IM diagnoses as well as COVID‐19 diagnoses from specialist care since 2015 [26].

The NPR coverage is almost 100% for inpatient care, as data reporting is mandatory for all physicians, both private and publicly funded. The coverage for specialist outpatient care is almost 100% for data from public caregivers; however, some data from private caregivers are missing [27]. According to Swedish laws and regulations, inpatient data reporting is mandatory for all physicians, both private and publicly funded. SmiNet, the national register of notifiable communicable diseases managed by the Public Health Agency of Sweden, was used to identify all individuals with a positive SARS‐CoV‐2 polymerase chain reaction (PCR) test. The Swedish Intensive Care Register provided dates of intensive care unit (ICU) admissions due to COVID‐19 since January 1, 2020. The NVR was used to obtain COVID‐19 vaccination dates.

### 2.2 Inclusion and Exclusion Criteria

The cohort in the current analysis comprises all individuals aged between 3 and 100 years who were resident in Sweden on January 1, 2020 (*N* = 9 978 860). Diagnosis of COVID‐19 among infants is unusual, and data for individuals over the age of 100 years is very limited; thus, a minimum age of 3 years and a maximum age of 100 years at baseline were set as inclusion criteria. Exclusion criteria were having an infectious mononucleosis diagnosis prior to the study entry date. Missing data were considered as an exclusion criterion, but no exclusions were necessary for this reason.

### 2.3 Exposure

Exposure definitions comprised positive SARS‐CoV‐2 PCR test results available for the entire country and the International Classification of Diseases, version 10 (ICD‐10) diagnosis codes U07.1 or U07.2 for specialist inpatient and outpatient care in the NPR. Exposure was modeled as a time‐dependent variable, coded 0 during periods with no recorded COVID‐19 infection, 1 from the first date of a positive SARS‐CoV‐2 PCR test from SmiNet (indicating “less severe” exposure), and 2 from the first date of hospital admission with COVID‐19, including intensive care (indicating “more severe” exposure). Each individual's follow‐up time was allocated based on these transitions, with the exposure remaining at the highest level reached until the end of follow‐up.

### 2.4 Outcome Assessment

The outcome was defined as a primary or secondary diagnosis in specialist outpatient or inpatient hospital care of infectious mononucleosis caused by EBV, identified using ICD‐10 code B27.0. In Sweden, the procedure for diagnosing infectious mononucleosis (ICD‐10 code B27.0) can vary. A patient with clinical signs of EBV infection is tested for heterophile antibodies or positive serology (i.e., IgM and low level of viral capsid antigen [VCA] IgG and negative EBV nuclear antigen [EBNA], and sometimes followed up by a test 4–6 weeks later to identify seroconversion, or a negative rapid test for Group A streptococci. It is less likely that the diagnosis is based on clinical symptoms alone, as testing in Sweden is easily accessible. The study population was followed from January 1, 2020, to the earliest diagnosis of IM due to EBV, date of emigration, death, or November 30, 2022, whichever occurred first.

### 2.5 Covariates

The Charlson comorbidity index (categorized as 0, 1, 2, ≥ 3) was created using NPR data before the start of follow‐up [28]. Other potential confounding factors included were sex, region of birth (Africa, Asia, European Union excluding Nordic countries, Europe excluding European Union and Nordic countries, North America, Nordic countries excluding Sweden, Oceania, former Soviet Union, Sweden, South America, and other), birth year (1920–1940, 1941–1960, 1961–1980, 1981–2000, 2001–2016), and Swedish healthcare region (North, South, Stockholm, South‐East, Uppsala‐Örebro, West, and other). Vaccination was identified from the NVR and included in a sensitivity analysis as a time‐varying variable, with exposure status changed from zero to one from the date of the first vaccination. Since children below the age of 12 were rarely vaccinated, an analysis including vaccination status was conducted, excluding children below the age of 12 years at baseline.

## 3 Statistical Analysis

The distribution of baseline characteristics (in January 2020) was tabulated by SARS‐CoV‐2 infection status (no infection, positive SARS‐CoV‐2 test only, and admission to hospital, including intensive care with COVID‐19) during the study period. Each individual is represented only once in an exposure category corresponding to the highest level of exposure reached by the end of follow‐up.

Age at EBV‐IM diagnosis and the duration between positive SARS‐CoV‐2 test only, COVID‐19 hospital admission, and EBV‐IM diagnosis were described by medians and interquartile ranges. Categorical variables were described using frequencies and corresponding percentages. Incidence rates of IM due to EBV per 100 000 person‐years, including 95% confidence intervals (95%CI), were estimated for each exposure category: males, females, and Charlson comorbidity index categories. Hazard ratios (HR) and 95% CIs for hospital‐diagnosed IM were calculated using Cox proportional hazards regression with the time‐varying SARS‐CoV‐2 exposure variable. Calendar time was used as the underlying timescale to control for pandemic waves and time‐varying variant strains of infection during follow‐up time. The adjusted models included sex, year of birth in categories indicating age, Swedish healthcare region, region of birth, and Charlson comorbidity index.

Additional analyses included stratification by sex, age at baseline, and study time before and after July 1, 2021, to reflect the shift in dominant variants around the time when the alpha variant was no longer dominant and was replaced by the delta and omicron variants. The proportional hazards assumption was tested using the Schoenfeld residual test, and no violation was found. Two‐sided *p* < 0.05 and 95% CIs not including 1.00 were considered statistically significant. All analyses were performed using Stata SE 18.0 (StataCorp LLC).

### 3.1 Sensitivity Analyses

In one sensitivity analysis, we excluded individuals (*n* = 4) who were diagnosed with IM due to EBV during their hospital stay for COVID‐19 to address potential surveillance bias. Another sensitivity analysis was conducted among comorbidity‐free individuals to explore whether only those with other diseases were at higher risk of infectious mononucleosis.

The final sensitivity analysis involved further adjustment for vaccination status, also as a time‐varying variable.

## 4 Results

Among 9 981 915 individuals aged 3–100 years, a total of 9 978 860 study participants remained after excluding 3055 individuals with an infectious mononucleosis diagnosis prior to the study entry date. None of the remaining individuals had missing data on covariates. Overall, 21.6% of the total study population remained unvaccinated during the study period, and most individuals (76.5%) received 2–5 vaccine doses. Table 1 shows baseline characteristics of the study population by SARS‐CoV‐2 infection status. Those who only tested positive were younger and with fewer comorbid diseases, compared to those without infection or admitted to hospital with COVID‐19. The patients admitted to the hospital were older and had more comorbidities, compared with the other two groups.

**Table 1:** Baseline characteristics of the study population of persons aged 3–100 years in Sweden (*N* = 9 978 860) in January 2020, by subsequent SARS‐CoV‐2 infection status by November 30, 2022.

Characteristic	No SARS‐CoV‐2 infection (*n* = 7 496 493)	SARS‐CoV‐2 positive test only (*n* = 2 370 362)	SARS‐CoV‐2 hospital admission (*n* = 112 005)
Age (years)	*n* (%)	*n* (%)	*n* (%)
3–10	715 759 (9.6)	205 764 (8.7)	792 (0.7)
11–20	851 149 (11.4)	323 058 (13.6)	1716 (1.5)
21–30	925 472 (12.4)	409 731 (17.3)	5171 (4.6)
31–40	908 072 (12.1)	443 977 (18.7)	7342 (6.6)
41–50	868 065 (11.5)	421 262 (17.8)	10 189 (9.1)
51–60	960 223 (12.8)	317 878 (13.4)	15 873 (14.2)
61–70	958 249 (12.8)	130 613 (5.5)	18 615 (16.6)
71–80	882 748 (11.8)	63 010 (2.7)	26 547 (23.7)
81–90	357 141 (4.8)	41 225 (1.7)	20 809 (18.6)
91–100	69 615 (0.9)	13 847 (0.6)	4951 (4.4)
Sex			
Male	3 855 670 (51.4)	1 099 895 (46.4)	62 224 (55.6)
Female	3 640 823 (48.6)	1 270 467 (53.6)	49 781 (44.5)
Charlson comorbidity index			
0	6 559 485 (87.5)	2 165 317 (91.4)	62 852 (56.1)
1	358 613 (4.8)	101 760 (4.3)	12 965 (11.6)
2	372 363 (5.0)	71 966 (3.0)	16 978 (15.2)
3 or more	206 032 (2.8)	31 319 (1.3)	19 210 (17.2)
Swedish healthcare region			
North	668 325 (8.9)	180 129 (7.6)	8150 (7.3)
South	1 335 010 (17.8)	436 272 (18.4)	16 196 (14.5)
Stockholm	1 723 229 (23.0)	554 058 (23.4)	31 728 (28.3)
South East	783 477 (10.5)	229 941 (9.7)	11 222 (10.0)
Uppsala‐Örebro	1 500 285 (20.0)	502 305 (21.2)	20 486 (18.3)
West	1 350 365 (18.0)	461 078 (19.5)	17 052 (15.2)
Other	135 802 (1.8)	6579 (0.3)	7171 (6.4)
Region of birth			
Africa	180 233 (2.4)	48 189 (2.0)	3043 (2.7)
Asia	569 804 (7.6)	200 426 (8.5)	12 765 (11.4)
European Union excluding Nordic countries	297 707 (4.0)	77 306 (3.3)	4408 (3.9)
Europe, excluding the European Union and the Nordic countries	185 393 (2.5)	76 233 (3.2)	5775 (5.2)
North America	31 684 (0.4)	9255 (0.4)	393 (0.4)
Nordic countries excluding Sweden	189 333 (2.5)	36 593 (1.5)	4980 (4.5)
Oceania	4997 (0.1)	1353 (0.1)	19 (0.0)
Former Soviet Union	4199 (0.1)	1066 (0.0)	121 (0.1)
Sweden	5 979 633 (79.8)	1 899 901 (80.1)	79 080 (70.6)
South America	52 118 (0.7)	20 763 (0.9)	1408 (1.3)
Other	1392 (0.0)	277 (0.0)	13 (0.0)

A total of 1424 individuals were diagnosed with IM due to EBV at a median age of 18 years (IQR: 15.5–22.8; range: 3.5–89.4). The overall rate of EBV‐IM was 5.0 (95% CI: 4.7–5.3) per 100 000 person‐years (Table 2). Among individuals with a positive SARS‐CoV‐2 PCR test only, 234 were subsequently diagnosed with EBV‐IM at a median age of 18.5 years (IQR: 16.6–22.3; range: 5.8–65.4). The median time between a positive PCR test only and the diagnosis of EBV‐IM was 222 days (IQR: 106–382; range: 4–757).

**Table 2:** Incidence rates and hazard ratios (HR) with 95% confidence intervals (CI) for an association between SARS‐CoV‐2 status and diagnosis of infectious mononucleosis caused by Epstein‐Barr virus among persons aged 3–100 years in Sweden, January 1, 2020, to November 30, 2022.

	Cohort		Rate^b^	Unadjusted model		Adjusted model^c^	
	Total	Events	(95% CI)	HR (95% CI)	*p*	HR (95% CI)	*p*
Total	9 978 860	1424	5.0 (4.7–5.3)				
SARS‐CoV‐2^a^							
Not diagnosed	9 978 860	1176	4.6 (4.4–4.9)	Reference		Reference	
Positive test only	2 391 382	234	7.8 (6.9–8.9)	1.71 (1.47–1.98)	< 0.001	1.61 (1.39–1.88)	< 0.001
Hospital admission	108 095	14	10.5 (6.2–17.6)	2.27 (1.34–3.85)	0.002	5.71 (3.33–9.79)	< 0.001

^a^ SARS‐CoV‐2 was modeled as a time‐varying exposure; hence, the total number of observations in each category of SARS‐CoV‐2 exposure is greater than the total number of individuals.

^b^ Per 100 000 person‐years.

^c^ Adjusted for sex, Charlson comorbidity index, birth year (1920–1940, 1941–1960, 1961–1980, 1981–2000, 2001–2016), Swedish healthcare region (North, South, Stockholm, South East, Uppsala‐Örebro, West, other), and region of birth (Africa, Asia, European Union excluding Nordic countries, Europe excluding European Union and Nordic countries, North America, Nordic countries excluding Sweden, Oceania, former Soviet Union, Sweden, South America, other).

### 4.1 More Severe Exposure: Hospital Admission for COVID‐19

**Figure 1:**
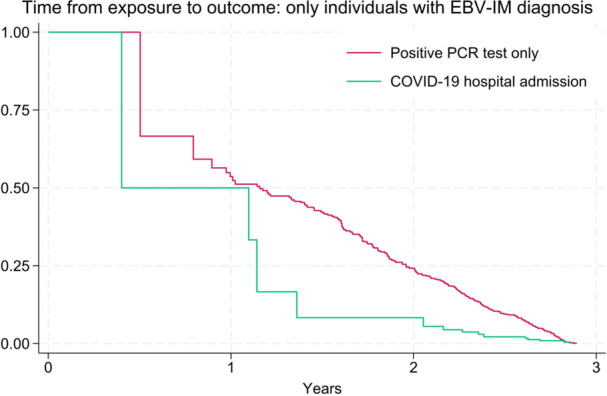
Time from SARS‐CoV‐2 positive test only (red; *n* = 234) and from the hospital admission with COVID‐19 (green; *n* = 14) to the outcome of infectious mononucleosis caused by Epstein‐Barr virus, among persons aged 3–100 years in Sweden, January 1, 2020, to November 30, 2022.

In the more severe exposure category, a total of 14 individuals were diagnosed with EBV‐IM following hospital admission for COVID‐19 at a median age of 56 years (IQR: 21.5–72.0; range: 8.2–79.4). The median time between hospital admission and diagnosis of EBV‐IM was 52 days (IQR: 2–206; range: 2–855). Figure 1 shows the time between the earliest date of a positive PCR test and the diagnosis of EBV‐IM, as well as the time between the first day of hospital admission with COVID‐19 and the diagnosis of EBV‐IM. Among 14 individuals admitted to the hospital with COVID‐19 who were diagnosed with EBV‐IM, 4 individuals were diagnosed with EBV‐IM during their hospital stay for COVID‐19 treatment. The remaining patients were diagnosed with EBV‐IM after discharge from the hospital, approximately 1 month to over 2 years after hospital admission for COVID‐19.

### 4.2 Less Severe Exposure: Positive PCR Test Only

A less severe SARS‐CoV‐2 infection, indicated by positive PCR test only, was associated with a higher risk of subsequent hospital diagnosis of EBV‐IM than for individuals without a COVID‐19 diagnosis in both the unadjusted model (HR: 1.71 [1.47–1.98]) and in the model adjusted for sex, Charlson comorbidity index, birth year, Swedish healthcare region, and region of birth (HR: 1.61 [1.39–1.88]) (Table 2). A higher magnitude association was observed in those with more severe infection, indicated by hospital admission with COVID‐19 (HR: 2.27 [1.34–3.85]). The magnitude of the positive association was larger in the adjusted model (HR: 5.71 [3.33–9.79]) (Table 2). In the same model mutually adjusting for all variables included in the model, the risk of EBV‐IM was higher among females than males (adjusted HR: 1.27 [1.15–1.41]) and Charlson comorbidity index scores of 1, 2, and 3 or more were associated with a higher risk of EBV‐IM (HR: 1.54 [1.28–1.86]; HR: 3.66 [2.71–4.95]; HR: 7.93 [5.61–11.22], respectively), compared to 0 in the adjusted model (Table SI).

### 4.3 Stratified Analysis

In the sex‐stratified analysis, associations with EBV‐IM were found among both men and women for positive test only (HR: 1.32 [1.04–1.69] and HR: 1.88 [1.55–2.30], respectively) and for hospital admission (HR: 6.57 [3.44–12.55] and HR: 4.08 [1.51–11.04], respectively) (Table 3). In the age‐stratified analysis, a positive SARS‐CoV‐2 test only was statistically significantly associated with EBV‐IM (adjusted HR: 1.36 [1.15–1.61]) only between ages 11 and 30 years, although the point estimates in all other age categories were similar but not statistically significant, which may be in part due to reduced statistical power (Table 3). For hospital admission with COVID‐19, the association with EBV‐IM was of higher magnitude and statistically significant across all age categories, although the number of EBV‐IM diagnoses in individuals with hospital admission was low across all age strata, particularly for the youngest group (3‐ to 10‐year‐olds), leading to wide CIs (Table 3). When SARS‐CoV‐2 exposure was stratified by time before and after July 1, 2021, the association for both positive test only and hospital admission was of higher magnitude for COVID‐19 exposure after July 1, 2021 (Table 3).

**Table 3:** Hazard ratios (HR) with 95% confidence intervals (CI) for an association between SARS‐CoV‐2 status and diagnosis of infectious mononucleosis caused by Epstein‐Barr virus, by sex, age, and study period.

SARS‐CoV‐2^a^	Cohort		Unadjusted model		Adjusted model^b^	
	Total	Events	HR (95% CI)	*p*	HR (95% CI)	*p*
Total	9 978 860	1424				
Sex						
Male						
Not diagnosed	5 017 789	553	Reference		Reference	
Positive test only	1 116 132	86	1.43 (1.12–1.81)	0.004	1.32 (1.04–1.69)	0.024
Hospital admission	60 057	10	3.14 (1.67–5.88)	< 0.001	6.57 (3.44–12.55)	< 0.001
Female						
Not diagnosed	4 961 071	623	Reference		Reference	
Positive test only	1 275 250	148	1.91 (1.58–2.32)	< 0.001	1.88 (1.55–2.30)	< 0.001
Hospital admission	48 038	4	1.36 (0.51–3.64)	0.542	4.08 (1.51–11.04)	0.006
Age						
3–10 years						
Not diagnosed	922 315	150	Reference		Reference	
Positive test only	205 766	17	1.18 (0.70–1.99)	0.541	1.39 (0.80–2.39)	0.241
Hospital admission	770	1	17.76 (2.48–127.35)	0.004	19.54 (2.71–140.60)	0.003
11–30 years						
Not diagnosed	2 516 297	851	Reference		Reference	
Positive test only	731 992	190	1.42 (1.21–1.67)	< 0.001	1.36 (1.15–1.61)	< 0.001
Hospital admission	6698	4	2.98 (1.12–7.97)	0.029	3.43 (1.28–9.18)	0.014
31–50 years						
Not diagnosed	2 658 907	79	Reference		Reference	
Positive test only	869 481	18	1.23 (0.73–2.10)	0.438	1.40 (0.80–2.45)	0.232
Hospital admission	17 249	2	5.53 (1.35–22.57)	0.017	6.88 (1.67–28.32)	0.008
51–70 years						
Not diagnosed	2 401 448	58	Reference		Reference	
Positive test only	458 443	7	1.12 (0.51–2.50)	0.773	1.43 (0.63–3.24)	0.389
Hospital admission	33 648	3	5.83 (1.82–18.69)	0.003	6.54 (2.01–21.31)	0.002
71–100 years						
Not diagnosed	1 479 893	38	Reference		Reference	
Positive test only	125 700	2	1.64 (0.39–6.91)	0.502	1.77 (0.42–7.49)	0.439
Hospital admission	49 730	4	8.13 (2.85–23.20)	< 0.001	8.14 (2.83–23.40)	< 0.001
Study period						
Before July 1, 2021						
Not diagnosed	9 978 860	650	Reference		Reference	
Positive test only	1 047 671	26	1.37 (0.91–2.05)	0.128	1.57 (1.05–2.36)	0.029
Hospital admission	73 213	4	2.55 (0.95–6.84)	0.062	5.26 (1.94–14.29)	0.001
After July 1, 2021						
Not diagnosed	8 705 296	526	Reference		Reference	
Positive test only	2 351 316	208	1.78 (1.51–2.09)	< 0.001	1.61 (1.37–1.90)	< 0.001
Hospital admission	95 500	10	2.18 (1.17–4.08)	0.02	6.49 (3.42–12.34)	< 0.001

^a^ SARS‐CoV‐2 was modeled as a time‐varying exposure; hence, the total number of observations in each category of SARS‐CoV‐2 exposure is greater than the total number of individuals.

^b^ Adjusted for birth year (1920–1940, 1941–1960, 1961–1980, 1981–2000, 2001–2016), sex (male, female), Swedish healthcare region (North, South, Stockholm, South East, Uppsala‐Örebro, West, other), region of birth (Africa, Asia, European Union excluding Nordic countries, Europe excluding European Union and Nordic countries, North America, Nordic countries excluding Sweden, Oceania, former Soviet Union, Sweden, South America, other), Charlson comorbidity index (0; 1; 2; 3 or more). Each analysis excludes an adjustment variable directly corresponding to the stratification variable.

### 4.4 Sensitivity Analyses

In the sensitivity analysis excluding patients diagnosed with EBV‐IM during hospital admission for COVID‐19 (*n* = 4), the magnitude of an association was attenuated (adjusted HR: 4.04 [2.14–7.61]). In the second sensitivity analysis restricted to comorbidity free individuals, the HR for individuals who were admitted to hospital for COVID‐19 is higher (adjusted HR: 7.91 [3.93–15.94], *n* = 8), whereas the hazard ratio for those with a positive SARS‐CoV‐2 test remained similar (adjusted HR: 1.66 [1.42–1.96], *n* = 212), compared with the main findings. In the final sensitivity analysis, additional adjustment for vaccination status (the first vaccination prior to SARS‐CoV‐2 detection as a time‐varying covariate) did not alter the results notably (data not shown).

## 5 Discussion

In this national study, SARS‐CoV‐2 infection was notably associated with an increased risk for subsequent EBV‐IM. A positive PCR test without hospital admission, which occurred in a larger number of individuals, was associated with a higher risk of subsequent EBV‐IM, compared with individuals who did not have a registered positive PCR test for SARS‐CoV‐2 infection. Among those who were admitted to the hospital with COVID‐19, the magnitude of association was greater. In all stratified and sensitivity analyses, the pattern of associations remained similar.

While some studies reported positive EBV DNA in plasma of COVID‐19 patients and confirmed EBV reactivation by conducting serological tests for antibodies against VCA IgM and early antigen‐diffuse EA‐D IgG as well as antibodies against the latent EBNA‐1 [20, 22], there are only case reports of IM due to EBV in COVID‐19 patients, including in an immunocompromised patient [23] and a 4‐year‐old child [24]. We used national population data with sufficient power to detect rare outcome events and further explore associations by exposure severity. The difference in susceptibility to EBV‐IM by sex has been described [29]. In our analysis, although women had a slightly higher rate of EBV‐IM hospital diagnoses, COVID‐19 was associated with a higher risk of EBV‐IM diagnoses in both sexes, with higher magnitude relative risks for EBV‐IM associated with hospital admission for COVID‐19 among men. This is consistent with a Danish study reporting a higher EBV‐IM diagnosis rate in women but a higher proportion with hospital admission for EBV‐IM among men [29]. EBV‐IM was very rare before age 11 years, which is consistent with more severe acute IM resulting in hospital admission when it occurs in adolescence and older ages. COVID‐19 was associated with EBV‐IM throughout adulthood, indicating this outcome was not only present during adolescence when IM is most common [30]. EBV‐IM, particularly when diagnosed before age 30 years, has been demonstrated as a risk factor for diseases later in life, such as cancer [31], including Hodgkin's lymphoma [5], MS [32], and possibly inflammatory bowel disease [19]. The association of COVID‐19 with EBV‐IM even among those with less severe COVID‐19 is important as it signals immune perturbation that may lead to longer‐term sequelae due to EBV among those who experienced COVID‐19 that did not require hospital admission. Additional adjustment for having at least one vaccination against SARS‐CoV‐2 before infection had little effect on the results within the severity levels of COVID‐19. This indicates that protecting against more severe manifestations of COVID‐19 may be the most important role for the vaccine in this context.

The inflammatory mechanisms potentially underlying an association between COVID‐19 and IM are complex and incompletely understood. Inflammation is a key feature of COVID‐19, which has been characterized as a highly inflammatory disease with elevated levels of proinflammatory molecules, including caspase‐1, IL‐1β, IL‐6, IL‐18, and TNF‐ɑ, particularly in more severe manifestations [33]. Long‐term changes to the peripheral immune system lasting weeks to months in individuals recovering from mild to moderate or severe SARS‐CoV‐2 are likely to have implications for susceptibility to new infections [9]. Some previous studies reported that individuals with COVID‐19 may be at increased risk of new infections, such as bacterial or fungal [34]. It is also known that SARS‐CoV‐2 virus triggers the activation of the NLRP3 inflammasome, which leads to the generation of a variety of proinflammatory molecules, including IL‐1β and IL‐18, that can cause extensive inflammatory reactions as well as cell death from inflammation [35, 36]. Some studies have demonstrated that activated NLRP3 enables EBV to reactivate from quiescence in memory B cells and start its lytic cascade [37]. Obesity, diabetes, smoking, and age‐related immune system decline are associated with COVID‐19 disease severity and mortality and may contribute to hyperinflammation through continuous activation of the NLRP3 inflammasome [38]. This may be one of the mechanisms explaining a stronger association between hospital admission with COVID‐19 and risk of EBV‐IM, compared to the risk associated with positive SARS‐CoV‐2 test only. Although this is one of the potential pathways, there may be other mechanisms through which EBV is reactivated, such as through changes in immunity and inflammation regulation resulting from post‐COVID‐19 gut dysbiosis [39], whereas fluvoxamine may block EBV reactivation [40, 41]. Regardless of the robustness of our findings and use of prospectively collected data, some potential limitations should be considered. Only more severe EBV‐IM was identified in this study, as infections treated in primary care were not identified. It was not possible to differentiate between EBV reactivation and primary IM using the available data, although individuals with IM prior to the pandemic were excluded (many of whom would have been infected asymptomatically). The association may be explained, at least in part, by surveillance bias among patients diagnosed with IM during their hospital stay with COVID‐19. In a sensitivity analysis, we excluded individuals hospitalized for COVID‐19 at the time of the IM diagnosis, and the association remained, albeit with somewhat lower magnitude. Such surveillance bias is less likely an explanation for the raised risk of EBV‐IM following a positive PCR test for SARS‐CoV‐2 without hospital admission, and there was a longer average duration between SARS‐CoV‐2 infection and EBV‐IM in this group, further reducing the likelihood of surveillance bias. In addition, we adjusted for the Charlson comorbidity index to address possible surveillance bias when IM symptoms may have been discovered during the hospital visits for other conditions. The association between SARS‐CoV‐2 exposure of any severity and risk of EBV‐IM was observed among comorbidity‐free individuals, suggesting that susceptibility to EBV‐IM was not restricted to those with comorbid diseases. In this study, we identified hospital‐treated EBV‐IM, so more severe acute IM will be captured. Because of the severity of the outcome, hospital‐treated IM due to EBV, outcome misclassification is highly unlikely. However, misclassification of exposure in individuals with mild or asymptomatic COVID‐19 who did not seek healthcare or PCR testing (including those who self‐tested and did not report positive results), and were classified as “not diagnosed,” is entirely possible. This non‐differential misclassification could have potential consequences for the magnitude of risk estimates, and the reported rates will be lower than actually existed. There may have been subsets of the population who had less contact with healthcare, such that COVID‐19 would be much less likely to be diagnosed. This may result in a lack of information from some parts of the population that could, in theory, be more susceptible to infection, thus introducing bias to the estimates. Some people would have tested regularly because of their type of employment or for travel: this may have detected more with mild asymptomatic disease, potentially diluting the magnitude of association with EBV‐IM, if greater severity increases IM risk.

Other potential limitations include the difficulty of addressing changes in exposure and susceptibility over time. In Sweden, the COVID‐19 vaccination program started at the end of December 2020 after the approval of the Pfizer/BioNTech vaccine. By the end of August 2020, 0.8% (*n* = 84 521) of the total population were already diagnosed with the infection, with a further 0.03% (*n* = 2560) admitted to an ICU [42]. Adjustment for vaccination status as a time‐varying covariate did not notably alter the association between COVID‐19 and EBV‐IM. The SARS‐CoV‐2 viral variant responsible for each infection could not be identified. In an attempt to control for changing characteristics of the pandemic, we used the calendar period as the underlying timescale and stratified by time before or after July 1, 2021, as an approximate start of delta variant dominance, since July 1, 2021, followed by Omicron variants, but we could not identify each variant specifically. EBV‐IM was more notably associated with COVID‐19 during the latter period. However, exploring an association for each dominant variant (Alpha, Delta, Omicron BA.1 and 2, and Omicron BA.5) was not possible, as we could only make assumptions about specific variants based on the date of infection. Among the population with a positive PCR test only as a marker of less severe COVID‐19, we could not further define severity, likely resulting in a degree of heterogeneity of severity among those with a positive PCR test for SARS‐CoV‐2 only. While EBV is associated with some chronic diseases, there was limited follow‐up time to detect diseases such as Hodgkin's lymphoma or multiple sclerosis due to the typically long duration between infection and clinical onset. We previously reported the association of hospital‐treated COVID‐19 with demyelinating diseases of the central nervous system [43]: none of those with hospital‐treated COVID‐19 and the rare outcome diseases had an intermediate diagnosis of EBV‐IM (data not shown). We did not examine chronic active EBV infection, as this is rare in Sweden, but more likely to occur in people from Asia, South and Central America, and Mexico [44].

In summary, our results show an association between COVID‐19 and subsequent EBV‐IM in both exposure categories: among those who only had a positive SARS‐CoV‐2 test and among those admitted to hospital with COVID‐19. The association remained robust in sex‐stratified and sensitivity analyses.

## Author Contributions

S.M., K.F., A.H., F.N., and S.V. conceptualized and designed the study. F.N. and H.L. ordered and managed the register data. S.V. and A.H. conducted statistical analyses. S.V. drafted the first manuscript version. All authors critically revised the manuscript, contributed to the interpretation of the findings, and approved the final version of the manuscript.

## Conflicts of Interest

Scott Montgomery has received multiple sclerosis research grants and/or honoraria for advisory boards/lectures from Roche, Novartis, AstraZeneca, Merck, Teva, and IQVIA. Fredrik Nyberg owns AstraZeneca shares. Fredrik Nyberg and Huiqi Li report participation in a research project funded by Bayer (regulator‐mandated phase IV study), with funds paid to the University of Gothenburg, where they are employed (no personal fees) and with no relation to the work reported here. The other authors declare no conflicts of interest.

## Supporting information


**Supplementary Table I**. Incidence rates and hazard ratios (HR) with 95% confidence intervals (CI) for an association between sex, Charlson comorbidity index, birth year, Swedish healthcare region, and region of birth and diagnosis of infectious mononucleosis caused by Epstein‐Barr virus among persons aged 3‐100 years in Sweden 1 Jan 2020 – 30 Nov 2022. **Supplementary Figure I.** Data sources of variables used in this study.

## Data Availability

The details of the data used in this study can be found at: https://www.gu.se/en/research/scifi-pearl. Access to the data can be requested via the SCIFI‐PEARL research group. Ethical permission for this record linkage study was obtained from the Swedish Ethical Review Authority (2020‐01800 with subsequent amendments).
