# Supplementary material for: SARS‐CoV‐2 Infection Is Associated With an Increased Risk of Hospital‐Treated Infectious Mononucleosis due to EBV: National Register‐Based Cohort Study
Source: J Med Virol. 2025 Dec 29;98(1):e70787. doi: 10.1002/jmv.70787 (PMC12746539; doi:10.1002/jmv.70787)
Supplement: Supplementary file 1 — Supplementary Table I. Incidence rates and hazard ratios (HR) with 95% confidence intervals (CI) for an association between sex, Charlson comorbidity index, birth year, Swedish healthcare region, and region of birth and diagnosis of infectious mononucleosis caused by Epstein‐Barr virus among persons aged 3‐100 years in Sweden 1 Jan 2020 – 30 Nov 2022. Supplementary Figure I. Data sources of variables used in this study. [file JMV-98-e70787-s001.docx]

# Supplementary material to:

**SARS-CoV-2 infection is associated with an increased risk of hospital-treated infectious mononucleosis due to EBV: national register-based cohort study**

Snieguole Vingeliene, Huiqi Li, Helena Backman, Ruzan Udumyan, Johan Jendeberg, Gunlög Rasmussen, Martin Sundqvist, Marleen A H Lentjes, Katja Fall, Ayako Hiyoshi, Fredrik Nyberg, Scott Montgomery

**Supplementary Figure I.** Data sources of variables used in this study.

**National Board of Health and Welfare**

**Public Health Agency of Sweden**

**National Quality Register**

**Statistics Sweden**

**The National Patient Register**

**SmiNet**

**National Vaccination Register**

**Swedish Intensive Care Register**

**Total Population Register**

COVID-19

ICD-10 codes U07.1 and U07.2

Infectious mononucleosis due to Epstein-Barr virus, ICD-10 code B27.0, inpatient and outpatient diagnoses

Charlson Comorbidity Index variables

COVID-19 diagnoses and

positive SARS-CoV-2 polymerase chain reaction test

Vaccination dates

Dates of intensive care unit admissions due to

COVID-19

Age, sex, birth year, death

Region of birth

Swedish healthcare region

Migration dates

**Supplementary Table I**. Incidence rates and hazard ratios (HR) with 95% confidence intervals (CI) for an association between sex, Charlson comorbidity index, birth year, Swedish healthcare region, and region of birth and diagnosis of infectious mononucleosis caused by Epstein-Barr virus among persons aged 3-100 years in Sweden 1 Jan 2020 – 30 Nov 2022.

|  | **Cohort** |  |  | **Rate**^a^ |  | **Unadjusted model** |  |  | **Adjusted model^b^** |  |
| --- | --- | --- | --- | --- | --- | --- | --- | --- | --- | --- |
|  | **Total** | **Events** |  | **(95% CI)** |  | **HR (95% CI)** | **P** |  | **HR (95% CI)** | **P** |
| Total | 9 978 860 | 1424 |  | 5.0 (4.7-5.3) |  |  |  |  |  |  |
| Sex |  |  |  |  |  |  |  |  |  |  |
| Male | 5 017 789 | 649 |  | 4.5 (4.2-4.9) |  | Reference |  |  | Reference |  |
| Female | 4 961 071 | 775 |  | 5.5 (5.1-5.9) |  | 1.21 (1.09-1.34) | <0.001 |  | 1.27 (1.15-1.41) | <0.001 |
| Charlson comorbidity index |  |  |  |  |  |  |  |  |  |  |
| 0 | 8 787 654 | 1209 |  | 4.8 (4.5-5.1) |  | Reference |  |  | Reference |  |
| 1 | 473 338 | 125 |  | 9.3 (7.8-11.0) |  | 1.94 (1.61-2.33) | <0.001 |  | 1.54 (1.28-1.86) | <0.001 |
| 2 | 461 307 | 49 |  | 3.9 (3.0-5.2) |  | 0.82 (0.62-1.10) | 0.186 |  | 3.66 (2.71-4.95) | <0.001 |
| 3 or more | 256 561 | 41 |  | 7.0 (5.1-9.5) |  | 1.47 (1.08-2.01) | 0.015 |  | 7.93 (5.61-11.22) | <0.001 |
| Birth year |  |  |  |  |  |  |  |  |  |  |
| 1920-1940 | 601 902 | 6 |  | 0.4 (0.2-0.8) |  | 0.40 (0.17-0.92) | 0.031 |  | 0.15 (0.06-0.35) | <0.001 |
| 1941-1960 | 2 145 311 | 74 |  | 1.2 (1.0-1.5) |  | 1.22 (0.89-1.68) | 0.221 |  | 0.80 (0.57-1.11) | 0.186 |
| 1961-1980 | 2 614 517 | 75 |  | 1.0 (0.8-1.2) |  | Reference |  |  | Reference |  |
| 1981-2000 | 2 679 121 | 313 |  | 4.1 (3.6-4.5) |  | 4.09 (3.18-5.26) | <0.001 |  | 4.70 (3.65-6.06) | <0.001 |
| 2001-2016 | 1 938 009 | 956 |  | 17.0 (16.0-18.2) |  | 17.18 (13.58-21.73) | <0.001 |  | 18.23 (14.36-23.15) | <0.001 |
| Swedish healthcare region | |  |  |  |  |  |  |  |  |  |
| North | 856 604 | 137 |  | 5.5 (4.7-6.5) |  | 1.09 (0.90-1.33) | 0.380 |  | 1.14 (0.93-1.39) | 0.202 |
| South | 1 787 478 | 308 |  | 6.0 (5.3-6.7) |  | 1.18 (1.01-1.38) | 0.037 |  | 1.22 (1.05-1.43) | 0.011 |
| Stockholm | 2 309 015 | 338 |  | 5.1 (4.6-5.6) |  | Reference |  |  | Reference |  |
| Southeast | 1 024 640 | 124 |  | 4.2 (3.5-5.0) |  | 0.83 (0.67-1.02) | 0.07 |  | 0.86 (0.70-1.05) | 0.144 |
| Uppsala-Örebro | 2 023 076 | 289 |  | 4.9 (4.4-5.5) |  | 0.98 (0.83-1.14) | 0.762 |  | 1.01 (0.87-1.19) | 0.859 |
| West | 1 828 495 | 215 |  | 4.1 (3.6-4.6) |  | 0.80 (0.68-0.95) | 0.012 |  | 0.82 (0.69-0.98) | 0.025 |
| Other | 149 552 | 13 |  | 13.6 (7.9-23.5) |  | 2.83 (1.62-4.95) | <0.001 |  | 5.37 (3.03-9.50) | <0.001 |
| Region of birth |  |  |  |  |  |  |  |  |  |  |
| Africa | 231 465 | 9 |  | 1.4 (0.7-2.6) |  | 0.23 (0.12-0.45) | <0.001 |  | 0.22 (0.11-0.42) | <0.001 |
| Asia | 782 995 | 29 |  | 1.3 (0.9-1.9) |  | 0.22 (0.15-0.32) | <0.001 |  | 0.23 (0.16-0.33) | <0.001 |
| European Union excluding Nordic countries | 379 421 | 23 |  | 2.2 (1.5-3.3) |  | 0.37 (0.25-0.56) | <0.001 |  | 0.51 (0.34-0.77) | 0.001 |
| Europe excluding European Union and Nordic countries | 267 401 | 5 |  | 0.7 (0.3-1.6) |  | 0.11 (0.05-0.27) | <0.001 |  | 0.18 (0.07-0.43) | <0.001 |
| North America | 41 332 | 7 |  | 6.1 (2.9-12.8) |  | 1.04 (0.50-2.20) | 0.908 |  | 1.24 (0.59-2.60) | 0.576 |
| Nordic countries excluding Sweden | 230 906 | 10 |  | 1.6 (0.8-2.9) |  | 0.27 (0.14-0.50) | <0.001 |  | 0.61 (0.32-1.13) | 0.117 |
| Oceania | 6 369 | 2 |  | 11.5 (2.9-46.1) |  | 1.97 (0.49-7.89) | 0.337 |  | 2.31 (0.58-9.25) | 0.237 |
| Former Soviet Union | 5 386 | 0 |  | - |  | - | - |  | - | - |
| Sweden | 7 957 614 | 1336 |  | 5.9 (5.6-6.2) |  | Reference |  |  | Reference |  |
| South America | 74 289 | 3 |  | 1.4 (0.5-4.4) |  | 0.24 (0.08-0.75) | 0.014 |  | 0.41 (0.13-1.27) | 0.123 |
| Other | 1 682 | 0 |  | - |  | - | - |  | - | - |

^a^Per 100,000 person-years.

^b^Mutually adjusted for sex, Charlson comorbidity index, birth year (1920-1940, 1941-1960, 1961-1980, 1981-2000, 2001-2016), Swedish healthcare region (North, South, Stockholm, South East, Uppsala-Örebro, West, other), and region of birth (Africa, Asia, European Union excluding Nordic countries, Europe excluding European Union and Nordic countries, North America, Nordic countries excluding Sweden, Oceania, former Soviet Union, Sweden, South America, other), and further adjusted for SARS-CoV-2 status.
